# Association of Tai Chi and Square Dance with Cognitive Function in Chinese Older Adults

**DOI:** 10.3390/healthcare12181878

**Published:** 2024-09-19

**Authors:** Xiaoguang Zhao, Dongxue Liu, Jin Wang

**Affiliations:** 1 Faculty of Sports Sciences, Ningbo University, Ningbo 315211, China; zhaoxiaoguang@nbu.edu.cn (X.Z.); 2301040009@nbu.edu.cn (D.L.); 2 Research Academy of Grand Health, Ningbo University, Ningbo 315211, China

**Keywords:** Chinese, cognitive function, older adults, square dance, Tai Chi

## Abstract

Objective: This study explores the association of Tai Chi and square dance with cognitive function and compares the effects of the two fitness programs on cognitive function in Chinese older adults. Methods: A total of 1732 older people (aged 60 years and over) met the inclusion criteria from the 2018 Chinese Longitudinal Healthy Longevity Survey. Based on the frequency of participating in Tai Chi and square dance, older adults were divided into three groups: a Tai Chi group (*n* = 234), a square dance group (*n* = 345), and a control group (*n* = 1153). Cognitive function was measured using a modified Mini-Mental State Examination (MMSE). Participation in Tai Chi or square dance was investigated by asking the subjects to report how often they participated in the fitness programs. Results: Older adults in both the Tai Chi group and the square dance group had higher scores in all MMSE items, including orientation, registration, attention and calculation, recall, and language, compared to those in the control group. But there were no significant differences in any MMSE items between the Tai Chi group and the square dance group. Multiple regression analysis showed that participating in Tai Chi or square dance, age, educational level, and sex can predict cognitive function in older people. Conclusion: Our findings suggest that participating in Tai Chi and square dance are associated with better cognitive function, and Tai Chi and square dance have similar effects on cognitive function in the Chinese older population.

## 1. Introduction

China is facing a very fast pace of population aging and has the largest older population in the world. According to the Chinese national population census in 2020, the number of people aged 60 and older in China has reached 264 million, and the proportion of older people is projected to be 18.7% [1]. It is predicted that by 2050, people aged over 60 years will number 400 million (25% of the total population) in China, of whom approximately 150 million will reach 80 years and older [2,3]. With the extension of life expectancy, how to prevent disease and preserve good health has become an issue of great importance for older people in China.

Cognitive decline is common in the older population. It is reported that the prevalence of cognitive decline in China ranges from 8.88% to 18.80% for adults aged 60 to 80 years [4,5]. Cognitive decline has been considered a primary cause of cognitive impairment, including dementia and Alzheimer’s disease [6]. Most previous studies have demonstrated that cognitive decline is closely associated with a series of mental and physical functions [7,8,9]. Furthermore, multiple studies have also found that a lower level of cognitive function is highly related to an increased risk of death [10,11,12]. It can be inferred that cognitive decline and cognition-related diseases will place a huge burden upon the healthcare system and families. Therefore, it is crucially important to prevent or delay cognitive decline for older adults.

Factors that impact cognitive function can be classified into non-modifiable factors (e.g., ethnicity, heredity, age, gender, and chronic diseases) and modifiable factors (e.g., nutritional supplements, physical activity, drinking, and smoking). As a modifiable factor, increasing physical activity may be more applicable because of its positive effect on body functions [13,14]. To date, a wide variety of physical activity promotion programs, including Tai Chi and square dance, have been implemented by Chinese older adults.

In China, Tai Chi and square dance are two popular fitness programs, especially for middle-aged and older people. Tai Chi is the traditional Chinese physical exercise. It has been proven to have numerous beneficial effects on physical health, such as increased muscle strength and balance control [15,16], as well as decreased heart rate and blood pressure [17,18]. Furthermore, scholars also found that Tai Chi is also likely to be associated with mental and brain health [19,20]. Square dance is an aerobic activity that integrates different types of dance with music rhythms. Studies have revealed that there is a close association between square dance and physical health, immune function, depression, subjective well-being, and life satisfaction in older adults [21,22].

Although multiple studies have shown that Tai Chi and square dance may be effective to improve physical and mental health in older adults [23,24,25,26], there are relatively few studies on the relationship between the two fitness programs and cognitive function. Therefore, the purpose of this study was to determine the association of Tai Chi and square dance with cognitive function and to compare the effects of the two fitness programs on cognitive function in Chinese older adults aged 60 years and over. Conducting such a study may be beneficial to improve older adults’ cognitive functioning. According to previous studies [16,22], this study hypothesized that both Tai Chi and square dance are associated with cognitive function, and Tai Chi has a better effect on cognitive function than square dance in Chinese older adults.

## 2. Materials and Methods

### 2.1. Study Population and Data Source

**Figure 1:**
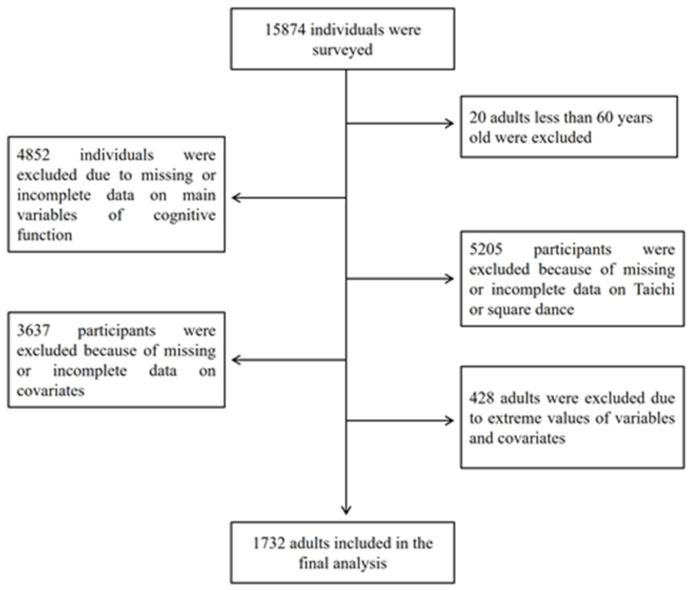
Data-filtering process.

This study used a cross-sectional design based on the national wave of the Chinese Longitudinal Healthy Longevity Survey in 2018 (CLHLS 2018), which was carried out between October 2017 and July 2019. The CLHLS data are available online on its official website (http://opendata.pku.edu.cn/dataverse/CHADS, accessed on 7 July 2024). In the CLHLS 2018, a face-to-face interview for 15,874 participants from 22 provinces in China was carried out by adopting an internationally compatible questionnaire on family, health, disability, behavioral, and demographic and socioeconomic risk factors. The inclusion criteria for the study participants were designed based on the purpose of this study as follows: (1) participants aged 60 years and older; (2) complete data on variables of cognitive function and participating in Tai Chi or square dance or not; (3) complete data on covariates such as age, sex, marital status, and educational level; and (4) values of variables and covariates in the normal range (values greater than two standard deviations around the mean were removed). As a consequence, 1732 older people were selected from 15,874 participants in the CLHLS 2018 according to the inclusion criteria (Figure 1). It should be noted that, since there were fewer than 30 respondents participating in both Tai Chi and square dance, they were removed from the final analysis. The CLHLS 2018 has been examined and approved by the Research Ethics Committee of Peking University (approval number: IRB00001052–13074). A written informed consent form was obtained from all the participants.

### 2.2. Tai Chi and Square Dance

Whether those who were interviewed participated in Tai Chi and square dance or not was investigated by asking the interviewees to report how often they participated in the fitness programs. Based on the frequency of participating in Tai Chi and square dance, older adults were divided into three groups: Tai Chi group, square dance group, and control group. In both the Tai Chi and square dance groups, the participants participated in the fitness programs at least one time per week. However, in the control group, the participants rarely engaged in Tai Chi or square dance (less than once per month).

### 2.3. Cognitive Function

The Mini-Mental State Examination (MMSE) is one of the most widely used instruments in the assessment of cognitive function. In this study, a Chinese version of the MMSE was adopted to evaluate the cognitive function of older people. In order to facilitate Chinese older persons’ responses and understanding, the CLHLS researchers modified the MMSE. The Chinese version of modified MMSE that has been frequently used in previous studies has been reported to possess good validity and reliability [27,28,29]. The modified MMSE includes five dimensions of cognitive function, such as orientation (e.g., what is the name of this county or district?), registration (e.g., repeat a sequence of three objects: table, apple, and clothes), attention and calculation (e.g., USD 20 − USD 3 = ?, USD 20 − USD 3-USD 3 = ?, USD 20 − USD 3 − USD 3 − USD 3 = ?, etc.), recall (e.g., repeat the three objects that were provided a while ago), and language (e.g., repeat an old Chinese saying: what you sow is what you get). The total MMSE scores range from 0 and 23. Higher scores represent better cognitive ability and vice versa. The Cronbach’s alpha in our study was 0.78 for the modified MMSE.

### 2.4. Covariates

Several covariates, including age, sex, anthropometry, co-residence, marital status, current smoker and drinker, occupation before age 60, and educational level, were measured in this study. Age was divided into four groups: 60–69 years, 70–79 years, 80–89 years, and ≥90 years. Anthropometry consisted of body height, weight, and body mass index (BMI). Co-residence was classified into “with household members”, “alone”, and “in an institution”. Marital status was classified into “married and living with spouse”, “separated”, “divorced”, “widowed”, and “never married”. If participants smoked or drank at that time, they were considered as current smokers or current drinkers. Occupation before age 60 consisted of governmental, institutional or managerial personnel, professional and technical personnel, agriculture, forestry, animal husbandry or fishery worker, self-employed, houseworker, etc. Educational level, as a continuous variable, was recorded in years of schooling.

### 2.5. Statistical Analysis

The data in SPSS format from the CLHLS 2018 dataset were obtained from its official website. This cross-sectional study first used descriptive statistics to present the characteristics of the participants. Categorical data were shown as percentages (%) and numerical data were expressed as mean with standard deviation (SD). Second, analysis of covariance (ANCOVA) with Bonferroni post hoc multiple comparisons was employed to examine the association of Tai Chi and square dance with cognitive function adjusted by age, sex, marital status, and educational level, and the effects of the two fitness programs on cognitive function in older adults were compared. Finally, this study used multiple regression analysis to examine which components are linked with cognitive function. In the regression analysis, the dependent variable was set as the total MMSE score, and the independent variables were set as participating in Tai Chi or square dance and other covariates with *p* < 0.10 in the univariate analysis. Subsequently, the stepwise method was used to obtain variables that remained in the final model. This study used IBM SPSS software (SPSS Inc., Chicago, IL, USA) version 25.0 to process and analyze the data. In the statistical analysis, *p* < 0.05 was considered to be statistically significant.

## 3. Results

Table 1 displays the demographic characteristics and MMSE scores of 1732 involved participants. In this study, there were 403 (23.27%), 685 (39.55%), 386 (22.29%), and 258 (14.90%) individuals aged 60–69 years, 70–79 years, 80–89 years, and ≥90 years, respectively. Women accounted for 53.30% and men made up 46.70% of the sample, with a mean BMI of 22.30 ± 3.85 kg/m^2^. More than 78% of older adults lived with household members and more than 64% of them were widowed. There were 46.07% of the participants who worked in agriculture, forestry, animal husbandry, or fishery before age 60. More than 86% and 85% of participants were non-smokers and non-drinkers. The proportions of older adults who participated in Tai Chi and square dance were 13.51% and 19.92%, respectively. The mean educational level was 4.51 ± 4.76 years and the mean total MMSE score of 1732 participants was 20.79 ± 3.27.

**Figure A1:**
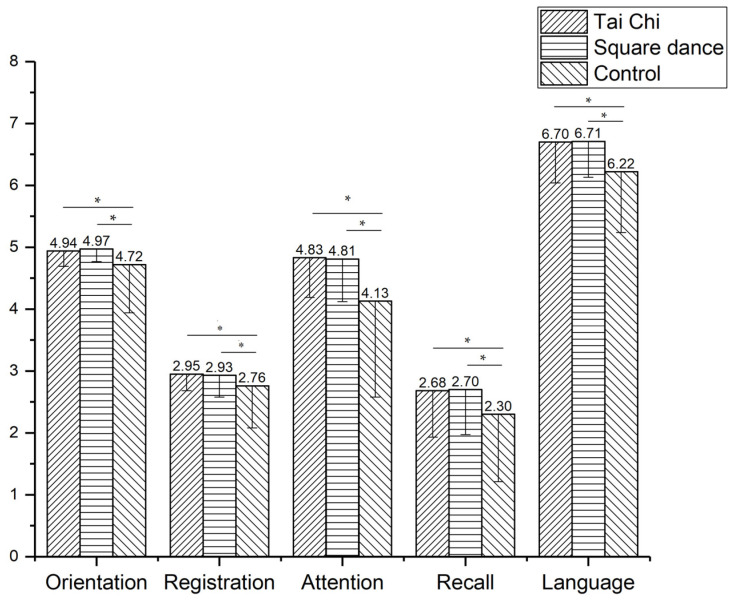
Comparisons of cognitive items among the 3 groups adjusted by age, sex, marital status, and educational level. * *p* < 0.05 compared to the control group using Bonferroni post hoc multiple comparisons test.

Comparisons of cognitive items among the Tai Chi group, square dance group, and control group are shown in Table 2 and Figure A1. ANCOVA showed statistically significant differences among the three different groups in all MMSE items, such as orientation, registration, attention and calculation, recall, language, and total MMSE score (all *p* < 0.01), which were adjusted by age, sex, marital status and educational level. Post hoc analysis demonstrated that older people in both the Tai Chi group and the square dance group had higher scores in all MMSE items compared to those in the control group (all *p* < 0.05). However, no significant differences in any MMSE items were observed between the Tai Chi group and the square dance group (all *p* > 0.05).

Table 3 describes the results of multiple regression analysis between the total MMSE score and participating in Tai Chi or square dance and other covariates. It was found that age (B = −0.57, *p* < 0.001), educational level (B = 0.11, *p* < 0.001), sex (B = −0.54, *p* = 0.002), and participating in Tai Chi or square dance (B = −0.36, *p* = 0.031) are the main components that can explain the total MMSE score. The mean explanatory power of the total MMSE score composed of participating in Tai Chi or square dance, age, educational level, and sex was 13% (R^2^) in Chinese older adults.

## 4. Discussion

This study aimed to determine the association of Tai Chi and square dance with cognitive function and to compare the effects of the two fitness programs on cognitive function in Chinese older adults. The results showed significant differences in all MMSE items, such as orientation, registration, attention and calculation, recall, language, and total MMSE score, among the three different groups. Older adults in both the Tai Chi group and the square dance group had higher scores in all MMSE items compared to those in the control group. However, there were no significant differences in any MMSE items between the Tai Chi group and the square dance group. Multiple regression analysis displayed that participating in Tai Chi or square dance, age, educational level, and sex can predict cognitive function in older people. These findings suggest that participating in Tai Chi and square dance are associated with better cognitive function, but there are no different effects on cognitive function between the Tai Chi and the square dance groups in the Chinese older population.

Physical activity plays an important role in the process of healthy aging [30,31]. Increasing physical activity may be beneficial for maintaining good cognitive and brain function in older adults [14]. A meta-analysis and systematic review noted that increasing physical activity through fitness programs might enhance the cognitive function of Alzheimer’s disease or slow down the decrease in cognition [32]. A randomized controlled trial showed that supervised moderate-intensity aerobic walking for 12 weeks might have potential cognitive benefits for older adults with schizophrenia [33]. Consistent with the results of previous studies, older people in both the Tai Chi group and the square dance group had greater scores in all MMSE items, including orientation, registration, attention and calculation, recall, and language, compared to those in the control group.

It is known that a tight adherence to fitness programs may play an important role in improving overall health and achieving successful aging in older adults [31]. Therefore, taking steps such as participants’ education, supervision, and communication and feedback to ensure that older individuals maintain these fitness programs over time is vital for the cognitive health of older people.

Both Tai Chi and square dance are very popular fitness programs in China which are particularly suitable for older people. A comparative study indicated that Tai Chi participants had better immune function and physical health than square dance participants, and Tai Chi and square dance had similar impacts on life satisfaction in older persons [22]. In the current study, there were no significant differences in cognitive function between the Tai Chi group and the square dance group. The current findings, combined with the previous results, indicate that there may be different effects on physical function (Tai Chi is superior to square dance) and no different effects on mental and cognitive function between Tai Chi and square dance. One possible explanation is that the effects on mental and cognitive function may depend on the physical activity’s intensity. Previous studies have pointed out that both Tai Chi and square dance are light-to-moderate-intensity exercise [34,35].

It follows from previous publications that frequency, duration, and participating years may have an important effect on outcomes of physical, mental, and cognitive health [30,36,37]. The current study divided the participants who participated in the fitness programs at least one time per week into Tai Chi and square dance groups. However, regarding the duration and participating years of these two fitness programs, they could not be made available because our study data were from the CLHLS and these factors were not measured in the CLHLS.

There are numerous factors that affect cognitive function in older adults. The factors can be categorized into two groups: non-modifiable factors and modifiable factors. Non-modifiable factors typically include ethnicity, heredity, age, gender, and chronic diseases [38,39,40]. Modifiable factors generally involve alcohol abuse, nutritional supplements, physical activity, and smoking [41,42,43]. In our study, the results of multiple regression analysis indicated that participating in Tai Chi or square dance, age, educational level, and sex can predict the cognitive function in older people. It is quite evident that only participating in Tai Chi or square dance is the modifiable factor. Hence, it can be argued that participating in Tai Chi or square dance frequently may delay or prevent cognitive decline in older adults.

The exact mechanisms behind the relationship between participating in Tai Chi or square dance and cognitive function in older people remain unclear. But it is possible to offer several explanations for the relationship. One plausible explanation is that both Tai Chi and square dance are light-to-moderate-intensity exercises, which may enhance cerebral blood flow, increase brain tissue metabolism, strengthen the central nervous system excitement, improve the establishment of brain neural networks, and therefore lead to adaptive changes in brain function and structure [44,45,46]. Moreover, participating in Tai Chi or square dance can lower psychological stress and increase interpersonal interaction and social support, which reduces stress-related psychiatric disorders and improves cognitive function [47,48].

There are several limitations to the current study. First of all, because this study used a cross-sectional design based on the CLHLS 2018 database, a precise cause–effect relationship between cognitive function and participating in Tai Chi or square dance in older adults cannot be established. Therefore, longitudinal studies are warranted to test the relationship. Moreover, this study used a modified MMSE to assess the cognitive function of older persons. Although the modified MMSE has been reported previously to possess good validity and reliability [27,28,29], there may be some differences between the original MMSE and the modified MMSE. Additionally, our study data were obtained from the CLHLS, and the duration and participating years of Tai Chi or square dance could not be obtained due to a lack of measurement. Finally, several covariates, including nutritional status, taking medications, disorders, and diseases, were not considered in this study, which may lead to biases in the interpretation of the study’s results.

Although there were several limitations, the present study still has advantages. To the best of our knowledge, it is the first study to compare the effects on cognitive function between Tai Chi and square dance in Chinese older adults. Furthermore, this study controlled a series of covariates, such as age, sex, marital status, and educational level, when exploring the association of participating in Tai Chi or square dance with cognitive function in older people.

## 5. Conclusions

The purpose of the study was to investigate the association of Tai Chi and square dance with cognitive function and to compare the effects of the two fitness programs on cognitive function. The findings suggest that participating in Tai Chi and square dance is associated with better cognitive function, and Tai Chi and square dance have similar effects on cognitive function in the Chinese older population.

The findings have significance in the disciplines of gerontology and geriatrics. Encouraging older persons to participate in Tai Chi or square dancing on a regular basis may be beneficial to their cognitive functioning. Future prospective research should look into whether Tai Chi or square dancing interventions improve cognitive performance in the older population.

## Figures and Tables

**Table 1:** The demographic characteristics and MMSE scores of involved participants (*n* = 1732).

Variables	Category	Numbers	Percentage (%) or Mean ± SD
Age			
	60–69 years	403	23.27
	70–79 years	685	39.55
	80–89 years	386	22.29
	≥90 years	258	14.90
Sex			
	Men	809	46.70
	Women	923	53.30
Anthropometry			
	Height, cm	1732	155.67 ± 9.99
	Weight, kg	1732	54.46 ± 12.52
	BMI, kg/m^2^	1732	22.30 ± 3.85
Marital status			
	Married and living with spouse	560	32.33
	Separated	39	2.25
	Divorced	4	0.23
	Widowed	1114	64.32
	Never married	15	0.87
Co-residence			
	With household member(s)	1358	78.41
	Alone	285	16.45
	In an institution	89	5.14
Occupation before age 60			
	Governmental, institutional, or managerial personnel	130	7.51
	Professional and technical personnel	210	12.12
	Commercial, service, or industrial worker	352	20.32
	Self-employed	38	2.19
	Agriculture, forestry, animal husbandry, or fishery worker	798	46.07
	Houseworker	142	8.20
	Military personnel	13	0.75
	Never worked	18	1.04
	Others	31	1.79
Current smokers			
	Yes	230	13.28
	No	1502	86.72
Current drinkers			
	Yes	251	14.49
	No	1481	85.51
Educational level		1732	4.51 ± 4.76
Participating in Tai Chi or square dance			
	Tai Chi	234	13.51
	Square dance	345	19.92
	None	1153	66.57
Modified MMSE score			
	Orientation	1732	4.80 ± 0.65
	Registration	1732	2.82 ± 0.59
	Attention and calculation	1732	4.36 ± 1.36
	Recall	1732	2.43 ± 1.01
	Language	1732	6.38 ± 0.91
	Total MMSE score	1732	20.79 ± 3.27

Note: BMI, body mass index; MMSE, Mini-Mental State Examination.

**Table 2:** Comparisons of cognitive items among the 3 groups adjusted by age, sex, marital status, and educational level.

Variables	Tai Chi (*n* = 234)	Square Dance (*n* = 345)	Control (*n* = 1153)	F Value	*p* Value ^a^
Orientation	4.94 ± 0.25 *	4.97 ± 0.20 *	4.72 ± 0.78	25.45	<0.001
Registration	2.95 ± 0.27 *	2.93 ± 0.35 *	2.76 ± 0.68	18.93	<0.001
Attention and calculation	4.83 ± 0.64 *	4.81 ± 0.69 *	4.13 ± 1.55	53.14	<0.001
Recall	2.68 ± 0.75 *	2.70 ± 0.73 *	2.30 ± 1.09	29.29	<0.001
Language	6.70 ± 0.66 *	6.71 ± 0.58 *	6.22 ± 0.98	57.92	<0.001
Total MMSE score	22.11 ± 1.76 *	22.11 ± 1.63 *	20.13 ± 3.65	76.66	<0.001

^a^ From the ANCOVA for comparing a difference among the 3 groups. * *p* < 0.05 compared to the control group using Bonferroni post hoc multiple comparisons test.

**Table 3:** Stepwise regression analysis between total MMSE scores and participating in Tai Chi or square dance and other covariates (*n* = 1732).

	B	SE	Beta	t	*p*
(Constant)	23.90	0.50		47.64	<0.001
Age	−0.57	0.11	−0.18	−5.05	<0.001
Educational level	0.11	0.02	0.16	5.92	<0.001
Sex	−0.54	0.17	−0.08	−3.15	0.002
Participating in Tai Chi or square dance	−0.36	0.16	−0.08	−2.16	0.031
	R^2^ = 0.13, Adjusted R^2^ = 0.12, F = 52.25, *p* < 0.001				

## Data Availability

Publicly available datasets were analyzed in this study. These data can be found here: [https://opendata.pku.edu.cn/dataverse/CHADS, accessed on 7 July 2024].
